# Visual and Topographic Improvement with Epithelium-On, Oxygen-Supplemented, Customized Corneal Cross-Linking for Progressive Keratoconus

**DOI:** 10.3390/jcm9103222

**Published:** 2020-10-08

**Authors:** Kazutaka Kamiya, Shunsuke Kanayama, Masahide Takahashi, Nobuyuki Shoji

**Affiliations:** 1 Visual Physiology, School of Allied Health Sciences, Kitasato University, Kanagawa 252-0373, Japan; 2 Department of Ophthalmology, School of Medicine, Kitasato University, Kanagawa 252-0374, Japan; istillremember03@yahoo.co.jp (S.K.); zavide96@gmail.com (M.T.); nshoji@kitasato-u.ac.jp (N.S.)

**Keywords:** customized CXL, CuRV, epi-on, oxygen, visual acuity, corneal topography, keratoconus

## Abstract

Customized cross-linking has been proposed as an alternative to conventional cross-linking in patients with progressive keratoconus, targeting greater flattening of the cone region and improved visual function. Epithelium-on cross-linking aims to reduce complications associated with epithelial removal, while the addition of oxygen aims to maintain treatment effect. Our study evaluates the combination of these novel treatment strategies to achieve a minimally invasive treatment targeting maximal functional outcomes. This prospective study included 42 eyes undergoing epithelium-on, accelerated, oxygen-supplemented, customized corneal cross-linking for progressive keratoconus. Outcome measures, including refraction, visual acuity, and corneal tomography were obtained at baseline and at 1, 3, and 6 months, and 1 year postoperatively. Logarithm of the minimum angle of resolution (logMAR) uncorrected visual acuity was significantly improved, from 0.87 ± 0.53 preoperatively, to 0.78 ± 0.56 1 year postoperatively (Wilcoxon rank sum test, *p* = 0.016). LogMAR best spectacle-corrected visual acuity was also significantly improved, from 0.19 ± 0.36 preoperatively, to 0.11 ± 0.33 postoperatively (*p* = 0.004). Manifest refractive cylinder was significantly decreased, from 4.50 ± 2.96 D preoperatively, to 3.27 ± 2.61 D postoperatively (*p* = 0.004). The baseline maximum keratometry (Kmax) was 53.04 ± 7.91 D, significantly flattening to 52.25 ± 7.31 D by 1 month, and remaining relatively stable at 1 year postoperatively (52.31 ± 7.50 D) (*p* < 0.001). No significant adverse events occurred in any eye. Epithelium-on, accelerated, oxygen-supplemented, customized corneal cross-linking is a promising new treatment approach, with reduced maximum keratometry, reduced astigmatism, and improved visual acuity at 1 year, with a favorable safety and patient comfort profile.

## 1. Introduction

Keratoconus is a bilateral corneal ectasia that results in progressive localized corneal thinning and steepening. Corneal cross-linking (CXL) is an established treatment to strengthen the corneal collagen stroma and to arrest the development of the keratoconus cone [1]. In the conventional epithelium-off (epi-off) CXL treatment protocol, the central epithelium is debrided, riboflavin photosensitizer eye drops are applied to the exposed corneal stroma until riboflavin flare is observed in the anterior chamber, and the corneal surface is uniformly irradiated with a 9-mm circular beam of Ultraviolet A (UVA) [2].

Conventional epi-off CXL has been shown to be an effective early intervention to stop keratoconus progression and preserve visual function [3]. While the primary goal of conventional CXL is disease stabilization, multiple studies demonstrate a degree of disease reversal in the form of post-treatment corneal flattening [4]. Customized cross-linking treatment protocols have been introduced to harness this flattening effect to reduce corneal irregularity and improve visual function [5]. In the customized cross-linking procedure, individual patient tomography is used to design the UVA treatment pattern to target the formation of more cross-link bonds in the weakened area of the cone [6,7]. The treatment is applied using a programmable UVA delivery device with integrated eye tracking to selectively activate the riboflavin photosensitizer in the targeted areas of the cornea [8]. Several controlled clinical studies have demonstrated greater corneal regularization in eyes treated with epi-off customized cross-linking as compared to conventional broad beam CXL, with greater flattening in the areas of steepest corneal curvature, compensatory steepening in the untreated surrounding cornea, and resultant gains in visual function [9,10,11].

Epithelium-on (epi-on) CXL has been proposed to reduce the risk profile of the procedure and to improve patient comfort by eliminating the epithelial debridement step [12]. The epithelium acts as a natural barrier to riboflavin, ultraviolet light, and oxygen, three key components of cross-linking photochemistry, which may limit the effectiveness of epi-on CXL [13,14,15]. Modifications to the epi-on treatment approach include the development of transepithelial riboflavin formulations [16,17] or iontophoresis to enhance delivery to the corneal stroma [18], pulsed, accelerated UVA irradiation protocols to reduce the rate of oxygen consumption [19,20], and most recently, the addition of supplemental oxygen at the corneal surface to increase the rate of oxygen diffusion [15,21,22].

The above treatment parameter modifications may theoretically be combined in an optimized cross-linking protocol for keratoconus using a transepithelial riboflavin formulation with supplemental oxygen and pulsed, accelerated, customized UVA irradiation to achieve a minimally invasive treatment approach while targeting maximal functional outcomes. The preliminary outcomes to evaluate this combination approach was recently reported by Mazzotta et al. [23] demonstrating flattening of corneal curvature, reduction in higher order aberrations, and improvement in visual acuity without significant adverse events in patients with stage 1 or 2 progressive keratoconus (Amsler–Krumeich stage) with 6-month follow-up. The present study evaluated this optimized treatment approach in patients with stage 1 through stage 4 progressive keratoconus in a Japanese population with 12-month follow-up. To our knowledge, this study is the first series to evaluate an optimized CXL protocol for a wide variety of progressive keratoconus, combining an epi-on technique with supplemental oxygen and pulsed, accelerated, customized UVA irradiation for a minimally invasive treatment approach targeting maximal functional outcomes.

## 2. Methods

This prospective observational study included 42 eyes with progressive keratoconus. Patients with progressive keratoconus were recruited from Kitasato University Hospital. Progression of keratoconus was defined as a ≥1.00 D increase in the maximum anterior curvature on corneal topography, or worsened corrected visual acuity accompanied by a ≥1.00 D increase in astigmatism confirmed in 2 or more examinations over the previous 12 months. The patients, who wore rigid gas permeable and soft contact lenses, were asked to stop wearing them for 3 and 2 weeks before this evaluation, respectively, in order to exclude the effect of wearing contact lenses. The study was approved by the Institutional Review Board of Kitasato University (C17-133), and followed the tenets of the Declaration of Helsinki. Written informed consent was obtained from all treated patients after explanation of the of the nature and possible consequences of the study.

Data were collected at baseline (preoperative) and at 1, 3, and 6 months, and 1 year. The examinations included subjective refraction, determination of uncorrected visual acuity (UCVA), and best spectacle-corrected visual acuity (BSCVA), slit lamp examination, and corneal tomography.

### 2.1. Cross-Linking Procedure

Table 1 summarizes the cross-linking parameters applied. A two-part transepithelial riboflavin-5-phosphate formulation (ParaCel Part 1 riboflavin 0.25% with benzalkonium chloride, ParaCel Part 2 riboflavin 0.22%, Avedro, Inc., Waltham, MA, USA) was used for the riboflavin application procedure. After installation of topical anesthetics and insertion of a lid speculum, a surgical spear sponge soaked with the part 1 solution was used to gently remove the mucin layer from the corneal surface, with care taken to avoid disruption of the epithelium. Two drops of the part 1 solution were instilled on the corneal surface every 60 s for 4 min. Excess part 1 solution was flushed from the eye with the part 2 solution. Additional drops of the part 2 solution were then applied every 30 s for 6 min. The corneal surface was gently flushed with approximately 5 mL of balanced salt solution (BSS).

**Figure 1:**
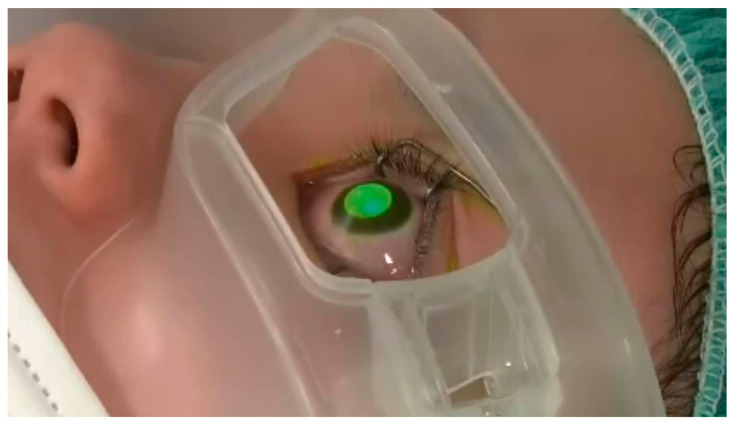
Application of the oxygen delivery goggles (Boost Goggles, Avedro Inc., Waltham, MA, USA). The difference map shows flattening of 3.0 D in the steep areas and steepening of 1.6 D in the previously flat areas.

Immediately after riboflavin application, oxygen delivery goggles (Boost, Avedro, Inc., Waltham, MA, USA) were fit to the patient face and connected to a bubble humidifier affixed to the medical grade oxygen source (Figure 1). The oxygen was turned on at a flow rate of 2.5 L/min. An oxygen meter was used to confirm ≥95% oxygen concentration within the goggles over the eye to be treated. A drop of topical anesthetic was applied to the corneal surface and the patient was aligned under the UVA system (Mosaic, Avedro, Inc., Waltham, MA, USA). The UVA system was turned on to deliver 365 nm UVA of 30 mW/cm^2^ irradiance pulsed (1 s on, 1 s off) according to the programmed treatment pattern. BSS was instilled on the cornea approximately every 2 min to maintain corneal hydration during UVA irradiation. The active eye tracking feature of the UVA system was used to maintain alignment throughout the treatment.

The customized UVA treatment pattern consisted of a graded pattern of three concentric ellipses of decreasing energy dose from center to periphery. Maximum energy dose applied was 15 J/cm^2^, consistent with previous studies of customized cross-linking using 30 mW/cm^2^ irradiance in patients with keratoconus [6,7,9,11]. The treatment pattern was centered on the location of the posterior float identified on corneal tomography, with the diameter of the treatment ellipses customized to cover the cone area, according the method previously described by Seiler et al. [10]. Following treatment, a bandage contact lens was inserted. Postoperative medications included preservative-free artificial tears, broad spectrum antibiotic drops, and steroidal anti-inflammatory drops.

### 2.2. Statistical Analysis

Statistical analyses were performed by using a statistical software (Bellcurve for Excel, Social Survey Research Information Co, Ltd., Tokyo, Japan). The normality of all data samples was first checked using the Kolmogorov–Smirnov test. Since all data did not fulfill the criteria for normal distribution, the Wilcoxon signed-rank test was used to compare the pre- and post-treatment. Unless otherwise indicated, the results are expressed as the mean ± standard deviation, and a value of *p* < 0.05 was considered statistically significant.

## 3. Results

### 3.1. Study Population

**Figure 2:**
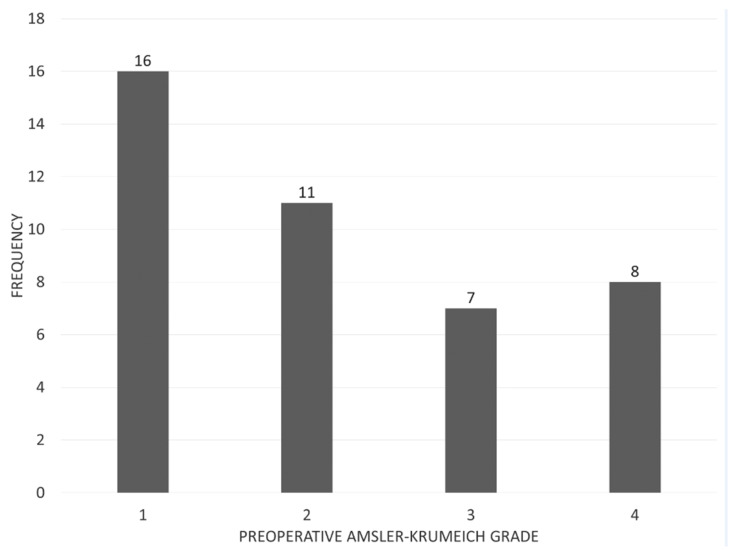
Distribution of preoperative Amsler–Krumeich staging.

A total of 42 eyes were treated with the epi-on customized cross-linking protocol (30 of males, 12 of females), aged 12 to 40 years (20.3 ± 5.2 years), and followed for 1 year. All eyes had documented progression of keratoconus, with disease severity graded as Amsler–Krumeich stage 1 to 4 at baseline (Figure 2). Preoperative and postoperative outcome measures are summarized in Table 2.

### 3.2. Visual Outcomes

LogMAR UCVA was significantly improved, from 0.87 ± 0.53 preoperatively, to 0.78 ± 0.56 1 year postoperatively (Wilcoxon rank sum test, *p* = 0.016). LogMAR BSCVA was also significantly improved, from 0.19 ± 0.36 preoperatively, to 0.11 ± 0.33 1 year postoperatively (*p* = 0.004). In terms of BSCVA, 50% of eyes (*n* = 21) gained 1 or more lines by 1 year postoperatively, with 26% of eyes (*n* = 11) gaining 2 or more lines. A total of 19% of the eyes (*n* = 8) lost 1 line of BSCVA, but no eyes lost 2 or more lines. We found no significant change in manifest spherical equivalent (−3.47 ± 3.87 D at baseline and −2.80 ± 3.02 D at 1 year) (*p* = 0.198), but a significant reduction of manifest refractive cylinder (4.50 ± 2.96 D at baseline and 3.27 ± 2.61 D at 1 year) (*p* = 0.004).

### 3.3. Corneal Topography

**Figure 3:**
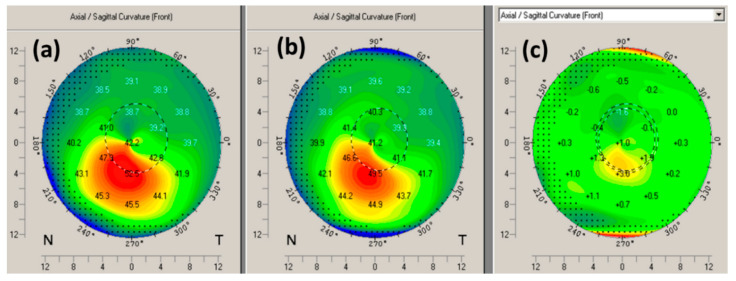
A representative example of corneal tomography at preoperative (**a**), 1-year postoperative (**b**), and the difference map showing the change in corneal tomography (**c**).

Qualitative analysis of axial topography difference maps indicated that the epi-on customized cross-linking procedure with supplemental oxygen resulted in improvement in overall corneal regularity (flattening of the cone region) in the majority of cases. A representative example of the change in corneal topography at 1 year after the procedure is shown in Figure 3. The difference map shows flattening of 3.0 D in the steep areas and steepening of 1.6 D in the previously flat areas.

**Figure 4:**
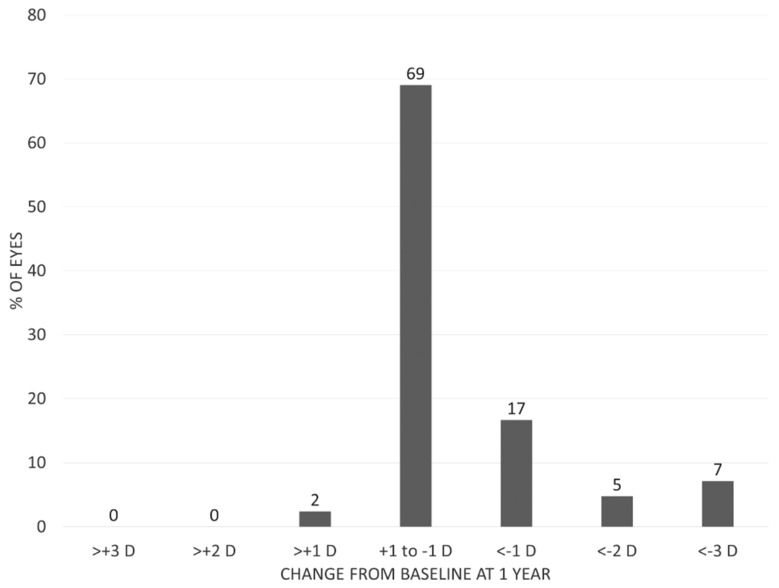
Distribution in changes in Kmax from preoperative values at 1-year post-procedure.

The distribution of changes in maximum keratometry (Kmax) from baseline to 1 year postoperatively is shown in Figure 4. The average baseline Kmax was 53.04 ± 7.91 D, significantly flattening to 52.25 ± 7.31 D by 1 month, and remaining relatively stable at 1 year postoperatively (52.31 ± 7.50 D) (*p* < 0.001). The majority of eyes (98%) had stable or flattened postoperative Kmax vs. baseline. Kmax steepening occurred only in 1 eye (2%), but this eye showed no change in BSCVA (20/12.5). In eyes with mild to moderate disease (Amsler–Krumeich grades 1 and 2), mean Kmax was 49.36 ± 5.03 D at baseline, flattening to 48.77 ± 4.66 D at 1 year (*p* = 0.014). In eyes with severe disease (Amsler–Krumeich grades 3 and 4), mean Kmax was 59.66 ± 7.95 D at baseline, flattening to 58.69 ± 7.52 D at 1 year (*p* = 0.013). In patients under the age of 18 (8 males, 3 females), aged 12 to 17 years (15.3 ± 1.8 years), mean Kmax was 53.94 ± 6.11 D at baseline, flattening to 53.17 ± 5.73 D at 1 year (*p* = 0.036).

Average mean keratometry (Km) was 50.62 ± 6.92 D, 50.22 ± 6.61 D, and 50.04 ± 6.69 D at baseline, 1 month, and 1 year postoperatively.

### 3.4. Safety

Patients reported mild to moderate discomfort for the first 24 to 48 h following the procedure, and a mild superficial punctate keratitis was observed in most eyes at 1 day postoperatively, which resolved by the 1-week postoperative visit. A transient postoperative haze, consistent with that reported after epithelium-off cross-linking, could be observed at the 1-week visit, presenting as a mid-stromal demarcation line by the 1-month postoperative visit.

The average endothelial cell density was stable at 1-year follow-up visits (2783 ± 224 cells/mm^2^ preoperatively and 2736 ± 280 cells/mm^2^ at 1 year) (*p* = 0.066). Similarly, there were no significant changes in thinnest corneal pachymetry (449 ± 42 µm preoperatively and 445 ± 40 µm at 1 year) (*p* = 0.330). No significant adverse events occurred in any eye.

## 4. Discussion

Our study evaluated a novel epi-on treatment approach aimed at maximizing the improvement in functional outcomes and minimizing the side effects of the CXL procedure in Asian population. The epithelium is a barrier to the absorption of riboflavin and metabolically consumes oxygen. Laboratory studies have indicated that the additional of supplemental oxygen to the corneal surface increases the rate of oxygen diffusion to the corneal stroma [15]. The addition of supplemental oxygen aimed to increase stromal oxygen levels for the cross-linking reaction. The customized UVA treatment pattern aimed to reduce corneal irregularity by specifically targeting the cone area. The short-term experience with this treatment approach demonstrated significant reduction in corneal curvature through 6 months with improvement in mean unaided and best corrected visual acuity in patients with mild to moderate keratoconus [23]. The results of our study revealed that the procedure slowed the progression of keratoconus, with flattening of Kmax observed through 1 year. This significant flattening of Kmax was observed in both mild to moderate and severe keratoconus. Furthermore, improvement of 1 or more lines of BSCVA was observed in 50% of patients.

In addition to supporting the findings of the Italian study, these outcomes are comparable to the outcomes of previous studies of epi-off customized cross-linking treatments, while applying the less invasive epithelium-on technique. Prior studies comparing epi-on and epi-off CXL treatment approaches suggest that while epi-on CXL may be effective at stabilizing keratometry in some patients with progressive keratoconus, the degree of corneal flattening achieved is reduced, relative to epi-off techniques [24]. Oxygen is an important mediator in cross-linking photochemistry, which can follow either an aerobic (Type I) or an anaerobic (Type II) pathway [14]. While cross-link formation is possible under both conditions, the aerobic pathway leads to more efficient generation of oxygen radicals compared to the anaerobic pathway. The addition of supplemental oxygen may improve the efficiency of epi-on cross-linking by increasing the rate of diffusion to the corneal stroma, such that oxygen is replenished faster than it is consumed [15]. This may be particularly relevant in epi-on protocols, where in addition to reduced oxygen bioavailability, the lipophilic epithelium also presents a physical barrier limiting the diffusion of riboflavin [13]. The efficacy of the epi-on treatment approach used in our study may be in part due to the addition of supplemental oxygen.

One limitation of our study is the absence of an epithelium-on control group without the addition of supplemental oxygen, which may have elucidated whether the favorable outcomes of this treatment approach were due to the advantages conferred by supplemental oxygen, the customized UVA treatment pattern, or a combination of both factors. However, the effectiveness outcomes in this study were objective, numeric parameters that were measured in the same eyes, at the same site, by the same clinicians, and on the same equipment both pre- and postoperatively; thus, patients’ preoperative measurements could reasonably be considered their own control. Analysis of corneal stromal demarcation lines via optical coherence tomography suggests that oxygen plays a role in deepening the treatment effect [23]. The presence of a double demarcation line demonstrated that the treatment dose impacted the depth of treatment, with a deeper demarcation line observed in the high dose area as compared to the low dose area [23]. Further, the overall depth of the demarcation line observed in that study was 30% deeper than in the author’s prior study of epi-off customized cross-linking without supplemental oxygen, demonstrating that the addition of oxygen contributed to deepening the treatment effect [23].

Our study included 11 pediatric patients between the ages of 12 and 17. Progression of keratoconus is more aggressive in pediatric patients [25], even when appropriate cross-linking treatment is applied [26,27]. In our study, significant flattening of Kmax relative to baseline was observed at 12 months with no significant adverse events, however, further follow-up and larger studies are needed to confirm the outcomes of the procedure in this treatment population.

Another potential area for future study could include the use of continuous (rather than pulsed) illumination in combination with supplemental oxygen. Comparative studies may also be useful in evaluating the present method of riboflavin application via transepithelial formulation vs. alternative delivery techniques, such as through a retention well or with iontophoresis.

The 1-year outcomes of our study demonstrate regularization of corneal curvature, improvement in visual function, and an absence of significant adverse events following the epi-on customized cross-linking treatment in patients with progressive keratoconus. We will continue to follow these patients to evaluate whether the cross-linking treatment was effective at stabilizing progression of keratoconus over the longer term and will report these findings in a subsequent publication. Although this longer-term evidence will be vital in assessing ongoing stability of treatment effect, it should be noted that changes in epithelial remodeling between 6 and 12 months after epi-off cross-linking are associated with underlying stromal change and are expected to be minimal [28].

## 5. Conclusions

In conclusion, epi-on, accelerated, oxygen-supplemented, customized corneal cross-linking is a promising new treatment approach to reduce corneal irregularity and improve visual function in patients with a variety of progressive keratoconus. In our series, we observed no significant progression of keratoconus, with reduced maximum keratometry, reduced astigmatism, and improved visual acuity at 1-year, with a favorable safety and patient comfort profile. No significant adverse events were observed. Large prospective randomized, controlled studies are needed to confirm the parameters contributing to the success of the procedure, and longer-term follow-up is needed to evaluate the stability of the treatment effect.

## Figures and Tables

**Table 1:** Epithelium-on (epi-on), accelerated, oxygen-supplemented, customized corneal cross-linking methods.

Epi-On, Accelerated, Oxygen-Supplemented, Customized Corneal Cross-Linking Methods	
Parameter	Variable
Treatment target	Keratoconus
Fluence (total) (J/cm^2^)	15
Soak time and interval (seconds)	(1) 4 min (q60s) + (2) 6 min (q30s)
Intensity (mW)	30
Treatment time (minutes and seconds)	16:40 (15 J), 11:06 (10 J), 8:00 (7.2 J)
Epithelium status	On
Chromophore	Riboflavin
Chromophore composition	(1) Benzalkonium Chloride/EDTA/Trometamol
	Hydroxypropylmethylcellulose with NaCl
	Phosphate buffered saline solution
	(2) With NaCl
	Phosphate buffered saline solution
Chromophore osmolality	Iso-osmolar
Chromophore concentration	(1) 0.25% (2) 0.22%
Light source	Mosaic (Avedro)
Irradiation mode	Pulsed (1 s on, 1 s off))
Protocol modifications	Oxygen-supplemented, customized
Protocol abbreviation in manuscript	Epi-on customized cross-linking

**Table 2:** Time courses of outcome measures in the study population.

All keratoconus (G1 to 4, *n* = 42)						Pre- vs. 1 year
Parameter	Preoperative	1 month	3 months	6 months	1 year	*p*-value
Thinnest pachymetry (µm)	449 ± 42	441 ± 38	441 ± 38	441 ± 38	445 ± 40	0.244
Kmax (D)	53.04 ± 7.91	52.25 ± 7.31	52.32 ± 7.33	52.37 ± 7.17	52.31 ± 7.50	<0.001
BSCVA (logMAR)	0.19 ± 0.36	0.10 ± 0.26	0.09 ± 0.32	0.13 ± 0.36	0.11 ± 0.33	0.004
UCVA (logMAR)	0.87 ± 0.53	0.78 ± 0.59	0.71 ± 0.57	0.75 ± 0.59	0.78 ± 0.56	0.016
Endothelial cell density (cells/mm^2^)	2801 ± 232				2772 ± 251	0.104
Mild to moderate keratoconus (G1 to 2, *n* = 27)						
Parameter	Preoperative	1 month	3 months	6 months	1 year	*p*-value
Thinnest pachymetry (µm)	464 ± 35	456 ± 25	454 ± 32	455 ± 28	458 ± 36	0.069
Kmax (D)	49.36 ± 5.03	48.84 ± 4.80	48.83 ± 4.75	49.10 ± 4.83	48.77 ± 4.66	0.014
BSCVA (logMAR)	0.06 ± 0.18	0.02 ± 0.17	0.00 ± 0.13	0.01 ± 0.17	0.02 ± 0.16	0.041
UCVA (logMAR)	0.72 ± 0.48	0.87 ± 0.54	0.87 ± 0.55	0.87 ± 0.56	0.78 ± 0.56	0.018
Severe keratoconus (G3 to 4, *n* = 15)						
Parameter	Preoperative	1 month	3 months	6 months	1 year	*p*-value
Thinnest pachymetry (µm)	421 ± 40	414 ± 43	417 ± 37	416 ± 42	422 ± 38	0.865
Kmax (D)	59.66 ± 7.95	58.38 ± 7.15	58.61 ± 7.04	58.26 ± 7.02	58.69 ± 7.52	0.013
BSCVA (logMAR)	0.42 ± 0.48	0.23 ± 0.33	0.26 ± 0.48	0.33 ± 0.50	0.29 ± 0.48	0.024
UCVA (logMAR)	1.15 ± 0.52	1.10 ± 0.50	1.02 ± 0.57	1.04 ± 0.61	1.09 ± 0.54	0.422

Kmax = maximal K reading; D = diopter; BSCVA = best spectacle-corrected visual acuity; UCVA = uncorrected visual acuity.
